# High Efficiency Video Coding Compliant Perceptual Video Coding Using Entropy Based Visual Saliency Model

**DOI:** 10.3390/e21100964

**Published:** 2019-10-02

**Authors:** Muhammad Zeeshan, Muhammad Majid

**Affiliations:** Department of Computer Engineering, University of Engineering and Technology, Taxila 47050, Pakistan; zeeshan249@gmail.com

**Keywords:** entropy, information maximization, high efficiency video coding, perceptual video coding, visual saliency

## Abstract

In past years, several visual saliency algorithms have been proposed to extract salient regions from multimedia content in view of practical applications. Entropy is one of the important measures to extract salient regions, as these regions have high randomness and attract more visual attention. In the context of perceptual video coding (PVC), computational visual saliency models that utilize the charactertistics of the human visual system to improve the compression ratio are of paramount importance. To date, only a few PVC schemes have been reported that use the visual saliency model. In this paper, we conduct the first attempt to utilize entropy based visual saliency models within the high efficiency video coding (HEVC) framework. The visual saliency map generated for each input video frame is optimally thresholded to generate a binary saliency mask. The proposed HEVC compliant PVC scheme adjusts the quantization parameter according to visual saliency relevance at the coding tree unit (CTU) level. Efficient CTU level rate control is achieved by allocating bits to salient and non-salient CTUs by adjusting the quantization parameter values according to their perceptual weighted map. The attention based on information maximization has shown the best performance on newly created ground truth dataset, which is then incorporated in a HEVC framework. An average bitrate reduction of 6.57% is achieved by the proposed HEVC compliant PVC scheme with the same perceptual quality and a nominal increase in coding complexity of 3.34% when compared with HEVC reference software. Moreover, the proposed PVC scheme performs better than other HEVC based PVC schemes when encoded at low data rates.

## 1. Introduction

Currently, the majority of information being communicated and shared on the Internet is in the form of multimedia. Images and videos captured from imaging and handheld devices possess an enormous amount of redundant information, which needs to be exploited for efficient transmission and storage. Traditionally, image and video coding techniques are developed with the aim to remove redundant information to reduce size, while preserving visual quality. The International Telecommunication Union (ITU) and the International Standards Organization (ISO) have developed a series of video coding standards over the last three decades. In 2010, the ITU video coding experts’ group (VCEG) and the ISO motion picture experts’ group (MPEG) created a joint collaborative team on video coding (JCT-VC) for the development of high efficiency video coding (HEVC), with the aim of achieving high compression gain [1]. Since the first draft of HEVC in April 2013, the research community has contributed to improving the performance of HEVC and its implementation on the hardware [2,3,4,5]. In Reference [2], a computationally scalable rate estimation algorithm is proposed that addresses the complexity issue associated with HEVC for encoding higher resolution videos. In Reference [3], FPGA-based hardware implementation of a video encoder is presented, which addresses the throughput of high resolution and high-quality videos in the entropy coding stage. A CABACbit rate estimation algorithm is implemented in FPGA and ASICbased hardware architecture, which exploits parallelism to improve the HEVC performance [4]. In Reference [5], FPGA and ASIC-based hardware architecture of HEVC intra encoder is presented that achieve a better performance in terms of computation workload reduction, BD-Rate and BD-PSNR.

Recently, researchers in the field of video coding have been focusing on reducing bit rate by utilizing the characteristics of the human visual system (HVS) and targeting higher quality for salient regions of video. This is going to benefit the network usability by reducing the required amount of bandwidth and helps to enhance the user experience. Psychovisual aspects of HVS have been employed in the video coding framework to remove perceptually redundant information. Video contains perceptually irrelevant information as humans generally focus on certain regions in a scene called region of interest (ROI). A perceptual video coding (PVC) scheme employs a visual saliency model to remove perceptually redundant information. Taking advantage of HVS characteristics, the PVC scheme screens out the perceptually irrelevant information present in the video. This improves the performance of video coding systems in terms of bit rate reduction, while maintaining the same perceived video quality. A visual saliency model can be integrated in a video coding framework in a variety of ways, which results in diversified PVC schemes. Generally, the PVC schemes are classified into two classes—pre-processing based PVC and embedded PVC [6].

Pre-processing based PVC schemes exploit HVS characteristics to modify the input video signal characteristics prior to encoding. In Reference [7], visual saliency based smoothing and enhancing is performed on the video frames before the encoding process. A foveation filter is incorporated at the pre-processing stage, which is modified by moving pattern classifier and the Hedge algorithm to suit the HVS mechanism. Spatial blurring is employed to remove the high-frequency contents from the image background, which represent the non-salient region [8]. As a result, the background is encoded at lower bit rate. In Reference [9], multiscale analysis and wavelet decomposition is employed to compute salient regions in video frames. Smoothing filters are applied to non-salient regions to remove high frequency content, which results in an improvement in compression efficiency. The overall performance of the pre-processing based PVC schemes is low because these methods are unable to fully utilize the video encoder characteristics. On the other hand, in embedded based PVC schemes, one or more functional blocks of the video coding framework are optimized, consistent with the HVS characteristics [10]. A visual saliency algorithm is employed to extract the perceptual features from video frames and adjust the encoder parameters accordingly. In Reference [11], HVS characteristics are utilized to optimize the distortion model of the HEVC encoder in accordance with the perceived image quality. A simplified perceptual rate-distortion optimization (RDO) procedure is adopted for the PVC scheme, which is influenced by the structural similarity index based divisive normalization scheme. In Reference [12], the PVC scheme adapts the scaling factor in the quantization block to the perceptual characteristics at macroblock level. In Reference [13], the frequency sensitivity of HVS is employed to improve the subjective quality of the video coding framework. The adaptive frequency weighting algorithm is utilized at the macroblock level to pick the frequency weighting factor for the quantization matrix.

In video coding, the data rate of the encoded bitstream is controlled by varying the quantization parameter (QP) value. As the QP value increases, the bitrate drops, but at the cost of visual quality. In PVC, several rate-control schemes have employed perceptual information for efficient resource allocation. In Reference [14], PVC architecture is proposed that computes a saliency map for each frame of input video and incorporate saliency information in video coding for non-uniform bit allocation. In Reference [15], the perceptual relevance of facial features in conversational videos is incorporated for rate control of HEVC. In Reference [16], the HEVC coding tree unit (CTU) and QPs are adaptively adjusted based on a hierarchical perception model of facial features in conversational videos. In Reference [17], a variable block-sized DCT kernel-based just-noticeable difference (JND) profile is proposed for PVC, where transform coefficients are suppressed according to perceptual distortion detection model. In Reference [18], a visual perception model is incorporated to extract texture and motion masking properties that optimized the rate-distortion optimization process in HEVC. Exploiting the fact that the HVS is not sensitive to the distortion of regions that have a complex texture and intense motion, it modifies the Lagrangian multiplier and QP value adaptively to the current CTU according to the video content. However, Lagrangian computation adds complexity, while selecting the best QP values. In Reference [19], an RDO scheme is adopted in HEVC reference implementation HM to select the best QP value in rate-distortion sense. The RDO scheme calculates a Lagrange multiplier before computing QP. However, in the RDO scheme, the perceptual relevance of each pixel in a frame is weighted uniformly [15], which results in needless equal bit allocation to ROI and non-ROI.

The moving objects in a video are the potential points to catch human attention. The spatial, as well as the temporal, characteristics of video have been utilized to generate a saliency map [20]. The spatiotemporal saliency map is then used for QP selection at coding unit level to guide bit allocation in the HEVC encoding framework. The JND model is employed in transformation and quantization blocks to phase out visually redundant information in HEVC [21]. For the transform skip mode in HEVC, the JND threshold is computed in the pixel domain by taking into account the luminance adaptation and contrast masking effects. For the transform non-skip mode, the transform domain JND threshold is estimated by considering the contrast sensitivity function. In Reference [22], the JND threshold based on perceptual redundancy in both luma and chroma channels is incorporated in HEVC at transformation and quantization stages to achieve bitrate saving and complexity reduction.

To the best of our knowledge, entropy based visual saliency models have not been incorporated in a video coding framework. Since entropy-based techniques have been effectively utilized to capture image features, it is therefore worth investigating the effectiveness of entropy-based visual saliency algorithms in a PVC framework. In this paper, a flexible and versatile HEVC compliant PVC framework is proposed that achieves bitrate reduction without degrading the perceived visual quality. An entropy based visual saliency algorithm is used to generate a saliency map at frame level. A binary saliency mask is created by thresholding the saliency map. A perceptual weight map is generated that identifies salient and non-salient CTUs. Different QP values are assigned to salient and non-salient CTUs in such a manner that the data rate is minimized while preserving the perceptual video quality. The major contributions of this work are:

1. Performance comparison of different entropy based visual saliency algorithms is presented for videos using a newly developed pixel-labeled ground truth.
2. Information maximization based visual saliency algorithm is incorporated in an HEVC framework.
3. An efficient algorithm to allocate quantization parameters for salient and non-salient CTUs is presented that minimizes the data rate while preserving the perceived quality.
4. The proposed entropy based PVC framework is evaluated objectively and subjectively and shows superior coding performance.

The rest of the paper is organized as follows. Section 2 describes the proposed HEVC compliant PVC framework. Section 3 presents the experimental results followed by the conclusion in Section 4.

## 2. Proposed Methodology

**Figure 1:**
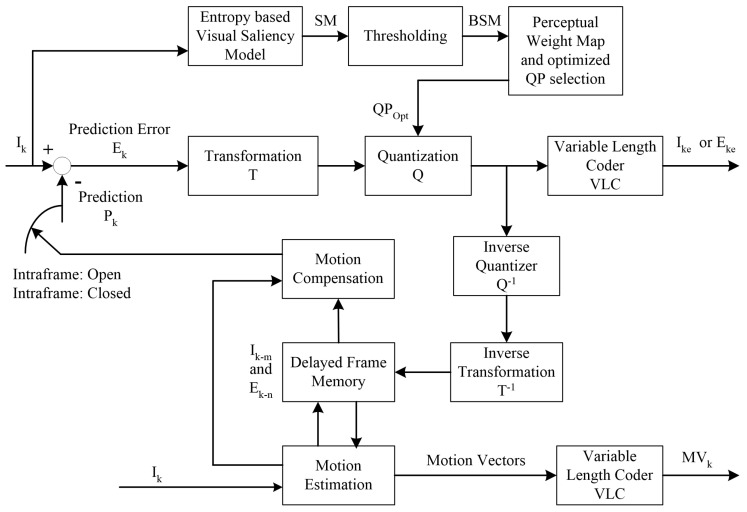
Block diagram of the proposed high efficiency video coding (HEVC) compliant perceptual video coding framework using entropy based visual saliency model.

The block diagram of our proposed HEVC compliant perceptual video coding framework using entropy based visual saliency model is shown in Figure 1. The saliency map of each frame generated by an entropy based visual saliency model is a grayscale image, which needs to be thresholded for a binary saliency mask. An optimal threshold value is obtained by comparing the generated saliency map with the human labeled ground truth to generate a binary saliency mask. The binary saliency mask is divided into CTUs in a similar fashion as in HEVC, which are categorized into salient and non-salient CTUs based on their perceptual relevance. An optimal QP value for salient and non-salient CTU is selected in such a way that the data rate is minimized while maintaining the perceived visual quality. The details of each block are presented in the following subsections.

### 2.1. Entropy Based Visual Saliency Model

Visual saliency has been the focus of psychologists, neurobiologists and computer scientists over the last few decades [23]. Computer scientists have developed numerous computational visual saliency algorithms, which aim at detecting the salient regions in an image. Computational visual saliency models find their applications in a broad spectrum of domains including remote sensing [24], watermarking [25], privacy [26], text detection [27], object recognition [28], multi-camera calibration [29], binocular vision [30], and video coding [31]. Generally, saliency detection techniques are categorized into bottom-up and top-down approaches. Bottom-up approaches are data-driven where the perception starts at the stimulus and top-down approaches are goal-driven where the saliency extraction is influenced by the task dependent cues. A great deal of research has focused on how human attention shifts while viewing a scene [32]. Attention theories [33,34] and earlier work on understanding human perception [35,36] suggest that the HVS is attracted to the regions in a scene that carry the maximum information [37].

Entropy has been extensively utilized in extracting and analyzing the salient regions from an image. It has been observed that image regions with high randomness attract more attention. A number of methods have been proposed that compute visual saliency from an entropy and information maximization perspective [38,39,40]. In this work, we selected four entropy based visual saliency models, namely attention based on information maximization [37], saliency and scale measures [41], entropy based object segmentation [42], and fuzzy entropy based multi-level thresholding [43] to generate a saliency map SM(p) from the input video frame. A brief description of each entropy based visual saliency model is as follows:

**1. Attention based on Information Maximization (AIM):** is based on Shannon’s theory and computes the self-information at each location of the frame [37]. AIM takes advantage of the fact that the HVS directs the attention mechanism to the most informative visual content in a scene.

**2. Saliency and Scale Measures (SSM):** capture the most salient features over different spatial locations and feature space [41]. Entropy maximization is used as a measure to identify salient regions in images. The scales are selected for each pixel location at which the entropy measure reaches its peak value. Degree of self-similarity is measured by using local descriptor statistics over a window of scales around the peak saliency measure.

**3. Entropy based Object Segmentation (EOS):** In EOS, a saliency map using local entropy as a feature is used, which represents the complexity and unpredictability of a local region [42]. The regions are considered salient if they have high complexity resulting in flat distribution, thus having higher entropy values.

**4. Fuzzy Entropy based Multi-Level Thresholding (FEMLT):** utilizes fuzzy entropy to segment an image’s foreground object from the background [43]. To segment foreground objects, the Shannon’s entropy of the input frame is computed at different thresholds, which are determined by normalized histogram. The entropy maximization approach is employed to select the optimum threshold, which is then used for segmentation.

### 2.2. Thresholding

The saliency map generated by the entropy based visual saliency model is a grayscale image, where pixel intensity specifies saliency relevance. The saliency map is normalized to range from 0 to 255 in such a way that the value 255 corresponds to the most salient pixels, while value 0 corresponds to the least salient pixels. The perceptual weight of a pixel increases as the intensity of saliency map increases. A binary saliency mask is generated by thresholding the grayscale saliency map as,

(1) $$BSM\left(p\right)=\left\{\begin{array}{cc} 1 & \mathrm{if}\;SM\left(p\right)>{\mathrm{Th}}_{o}\\ 0 & \mathrm{otherwise}, \end{array}\right.$$

where ${\mathrm{Th}}_{o}$ is the optimal threshold value. BSM(p) is a pixel-level binary mask, where pixel value 1 corresponds to salient pixel in a frame, while pixel value 0 corresponds to a non-salient pixel. The choice of an optimal threshold value ${\mathrm{Th}}_{o}$ to generate a binary saliency mask is critical as it influences deciding on the salient and non-salient regions and hence the encoding cost of the overall framework. A pixel-level accurate human labeled ground truth was required for comparison to select the optimal threshold value. Pixel-accurate labeling of salient objects within the frame was obtained through subjective experiment. Each frame was shown to 9 subjects and they were asked to label the salient region. Majority voting criteria was adopted to generate a single aggregated ground truth binary mask GTM(p) for each frame, where pixel value 1 corresponds to salient and 0 corresponds to non-salient regions. The steps involved in selecting the optimal threshold value were as follows:

1. Initialize the threshold vector $Th_{i}$ with *N* values as,
  (2) $$Th\left(1,i\right)=Min\left(SM\left(p\right)\right)+\left(i\times \frac{\left\{Max(SM(p))-Min(SM(p))\right\}}{N}\right),$$
  where Min(SM(p)) and Max(SM(p)) represents the minimum and maximum value of the saliency map generated by the visual saliency algorithm respectively. The number of threshold levels is represented by *N* and i=0,1,⋯,N−1.
2. Initialize a vector $F_{m}$ of size 1×N representing average F-measure values with all zeros.
3. Calculate the thresholded saliency map $TSM_{i}\left(p\right)$ of each video frame in the dataset at threshold value Th(1,i) as
  (3) $$TSM_{i}\left(p\right)=\left\{\begin{array}{cc} 1 & \mathrm{if}\;SM(p)>Th(1,i)\\ 0 & \mathrm{otherwise}, \end{array}\right.$$
4. Calculate the F-measure between the thresholded saliency mask $TSM_{i}\left(p\right)$ and human labeled ground truth binary mask GTM(p) for all video frames in the dataset.
5. Compute the average F-measure and store in the vector $F_{m}$ at ith position.
6. Repeat steps 3 to 5 for all threshold values.
7. Choose index from vector $F_{m}$ that gives maximum average threshold value as optimum threshold value.

### 2.3. Perceptual Weight Map and Optimized QP Selection

The binary saliency mask generated by thresholding is divided into CTUs in a similar way as done by HEVC reference software. CTUs are then categorized into two categories—salient and non-salient CTUs—based on their perceptual significance. The salient and non-salient pixels are quantified to mark the perceptual significance of each CTU. The percentage of salient pixels in a CTU is determined as,

(4) $$P_{CTU}=\frac{N_{s}}{N}\times 100,$$

where $N_{s}$ and *N* represent the number of salient pixels and total number of pixels in the CTU of binary saliency mask. The CTU based perceptual weight mask is obtained as,

(5) $$WM_{CTU}=\left\{\begin{array}{c} 0,\;P_{CTU}\leq \;50\%\\ 1,\;P_{CTU}>\;50\%, \end{array}\right.$$

As the proposed PVC scheme depends on the perceptual significance of CTU, therefore an optimized quantization parameter is required for each CTU based on their perceptual significance. CTUs that fall in the salient region attract more attention as compared to those CTUs which belong to a non-salient region. Therefore, to enhance the perceptual quality, an optimal criterion is required to assign QP values to different CTUs.

Let $QP_{d}$ be the default QP value for all CTUs in the frame. The CTU-based perceptual weight map categorizes CTUs into salient and non-salient CTUs. Then the optimized $QP_{o}$ values for non-salient CTUs are computed as,

(6) $$QP_{o}=QP_{d}+AF,$$

where AF represents the QP adjustment factor for non-salient CTUs. The value of AF depends on the saliency significance of a CTU and is selected in such a way to minimize the perceptual distortion at default quantization parameter i.e., $QP_{d}$. The procedure of selecting optimum QP for non-salient CTUs i.e., $QP_{o}$ depends on tolerated difference in perceptual quality i.e., ΔQ. The tolerated difference in perceptual quality shows the difference in average perceptual quality using the default QP and optimized QP. The computation procedure of selecting optimized QP for salient and non-salient CTUs is described in Algorithm 1. 
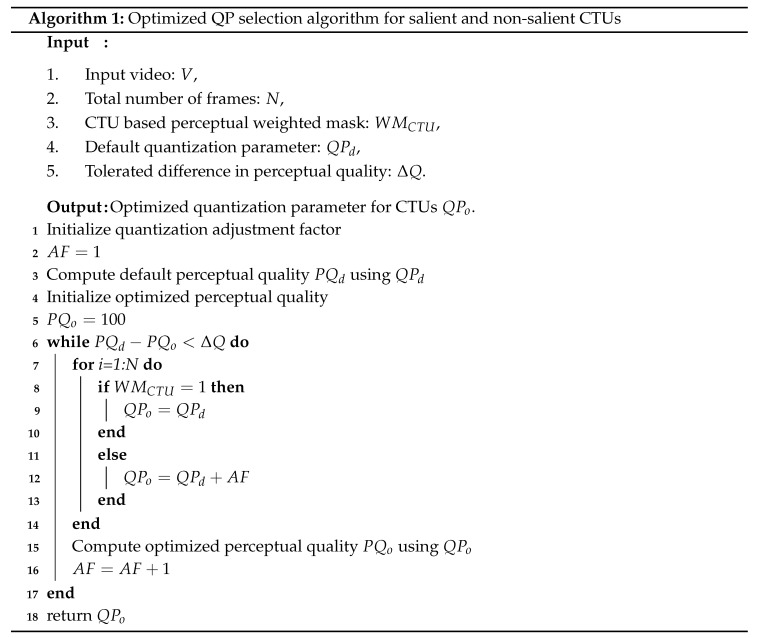


## 3. Experimental Results

Video content has a high impact on encoder performance, therefore test video sequences for HEVC are defined according to resolution, application domain and genre [1]. In this paper, sixteen test video sequences of class A, B, C, D, E, F and 4K were selected for the purpose of evaluation [44]. The selected video sequences cover a variety of resolutions, that is, 4K, HD 1080p, HD 720p, WVGA, WQVGA and frame rates, that is, 24, 30, 50, 60 and 120 frames per second (fps) and statistical features. The details of video sequences used in this paper are presented in Table 1. The experimental results are presented in two sections. In the first set of experiments, the performance of different entropy based visual saliency models is compared. In the second set of experiments, the best entropy based visual saliency model is incorporated in HEVC standard for the proposed PVC scheme, which is compared with HEVC reference software and other PVC schemes in an objective and a subjective manner.

### 3.1. Performance Comparison of Entropy Based Visual Saliency Models

In this set of experiments, the performance of four entropy based visual saliency algorithms (AIM [37], SSM [41], EOS [42], and FEMLT [43]) were compared in both quantitative and qualitative manners. The main aim of this comparison is to select the best entropy based visual saliency algorithm and optimum threshold value to generate a binary saliency mask based on a human labeled groundtruth binary mask. In this work, pixel-accurate labeling of salient and non-salient was adopted as it offers extensive and accurate evaluation as compared to rectangular bounding box based labeling [45]. For pixel-level groundtruth mask construction, 9 subjects were involved. The video frames were shown to subjects, which were instructed to precisely mark the salient objects at the pixel-level accuracy. The final groundtruth mask was obtained by applying majority voting criteria to remove labeling inconsistency.

**Figure 2:**
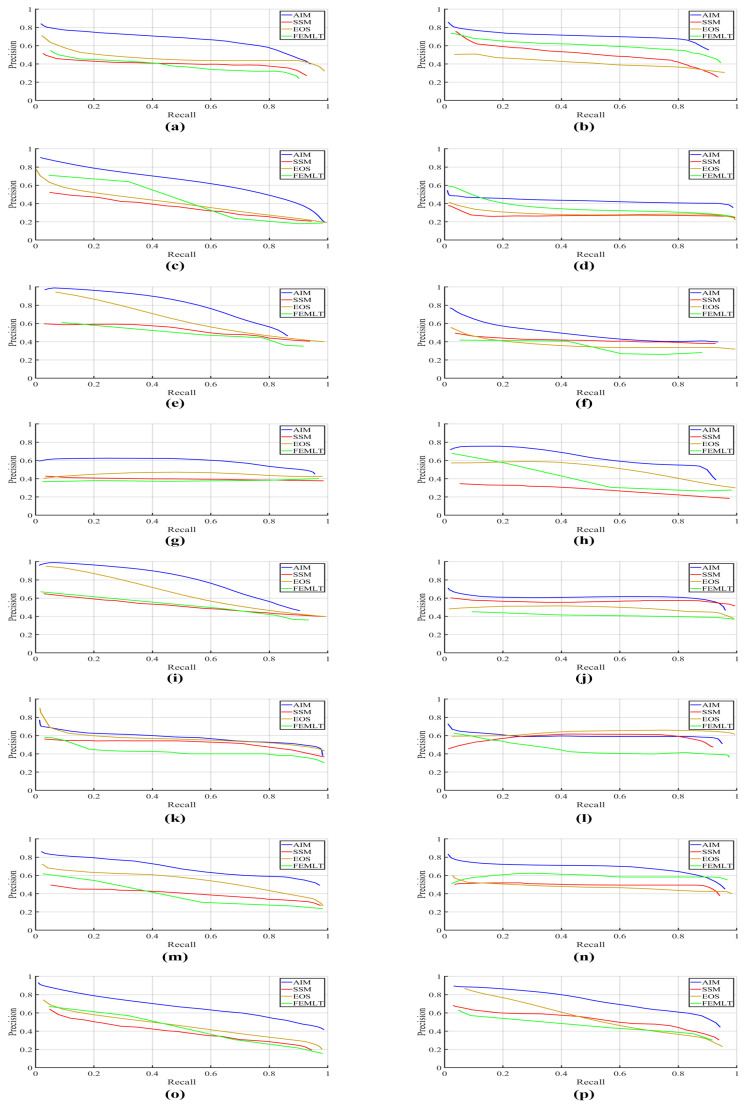
Precision-Recall curves of different entropy based visual saliency models for video sequences (**a**) Nebuta (**b**) SteamLocomotive (**c**) BasketballDrive (**d**) ParkScene (**e**) RaceHorses (**f**) BQMall (**g**) PartyScene (**h**) BasketballDrill (**i**) RaceHorses (**j**) BlowingBubbles (**k**) FourPeople (**l**) Johnny (**m**) BasketballDrillText (**n**) SlideShow (**o**) Bosphorus (**p**) Jockey.

**Figure 3:**
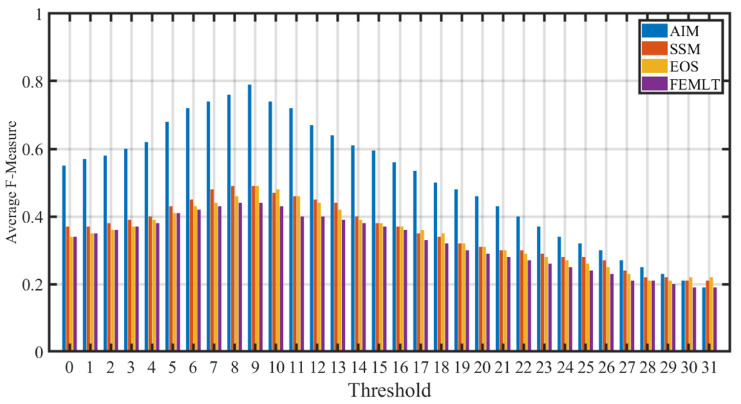
Performance comparison of different entropy based visual saliency algorithm in terms of average F-measure for different threshold values.

Precision-recall (PR) curves and F-measure were employed as metrics for the quantitative performance comparison of different visual saliency algorithms. Precision is the ability of a visual saliency model to label the non-salient pixels as non-salient, whereas recall is an ability of a visual saliency model to correctly mark the salient pixels as salient. F-measure presents the harmonic mean of precision and recall. The binary saliency maps are evaluated objectively to figure out the correspondence with the human-labeled groundtruth. The precision, recall and F-measure score varies with the change of threshold value, therefore the appropriate selection of threshold value is a critical issue to generate a binary saliency mask. The saliency map generated by each visual saliency algorithm was thresholded at 32 threshold values. For each threshold value, a corresponding binary mask was generated and the equivalent precision and recall were computed using a binary groundtruth mask. Figure 2 shows the PR curves of different entropy based visual saliency algorithms (AIM, SSM, FEMLT and EOS) computed over all 16 videos in the dataset. It is evident that the AIM visual saliency algorithm gives the best PR curve except for the Johnny video. This shows that the AIM visual saliency algorithm gives higher precision and recall values for majority of the videos. Figure 3 depicts the performance comparison of different entropy based visual saliency models in terms of average F-measure computed over all video sequences for different threshold values. A higher F-measure value indicates better performance of the visual saliency model when compared with the human labeled groundtruth binary mask. It is evident that AIM gives higher average F-measure values than SSM, FEMLT and EOS for all threshold values. Moreover, a maximum value of average F-measure achieved by AIM is at threshold value 9. The average precision, recall and F-measure values by different entropy based visual saliency algorithms, when compared with pixel-level binary groudtruth mask at $Th_{o}=9$, are shown in Table 2. It can easily be observed that high precision, recall and F-measure values are achieved by AIM as compared to SSM, EOS, and FEMLT based visual saliency algorithms.

**Figure 4:**
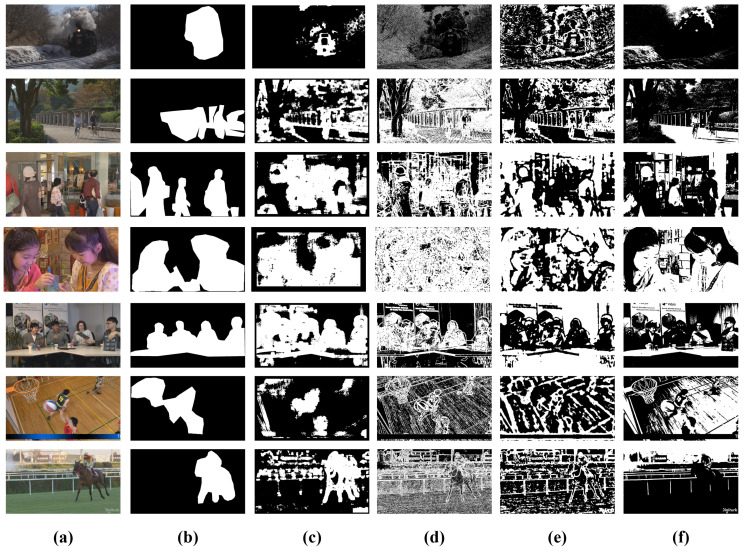
Qualitative comparison of different entropy based visual saliency algorithms (**a**) representative video frames, (**b**) aggregated pixel-level binary groundtruth mask (**c**) AIM (**d**) SSM (**e**) EOS (**f**) FEMLT, Row 1: SteamLocomotive (Class A), Row 2: ParkScene (class B), Row 3: BQMall (class C), Row 4: BlowingBubbles (class D), Row 5: FourPeople (class E), Row 6: BasketballDrillText (Class F), Row 7: Jockey (4K).

The qualitative comparison of salient regions detected by different entropy based visual saliency algorithms and groundtruth for representative frame from seven video sequences of class A, B, C, D, E, F, and 4K in the dataset at optimum threshold $Th_{o}=9$ is shown in Figure 4. We observed that the AIM visual saliency algorithm gives a better binary saliency mask after thresholding than SSM, EOS and FEMLT visual saliency algorithms, when compared with aggregated pixel-level binary groundtruth mask. The pixel-level binary groundtruth mask highlights salient and non-salient regions in the frame with white and black values, respectively. The salient pixels detected by AIM in Figure 4c coincide well with the groundtruth binary mask. Moreover, very few non-salient pixels are detected as salient. On the other hand SSM, EOS and FEMLT partially detect salient pixels as salient and majority of the non-salient pixels are also detected as salient, which is evident from Figure 4c–e. These qualitative results are also consistent with quantitative results as average precision, recall and F-measure achieved by AIM is much higher than the average precision, recall, and F-measure of the SSM, EOS and FEMLT models.

### 3.2. Perceptual Video Coding

To verify the effectiveness of the proposed PVC framework, the saliency model AIM was incorporated into the HEVC reference software HM 16.11 [46]. The AIM model was selected because it gives a better performance than other entropy based visual saliency algorithms.The saliency map of each frame is thresholded by using $Th_{o}=9$ to generate a binary saliency mask that is used to divide a frame into salient and non-salient regions. A perceptual weight map is computed, which indicates the perceptual significance of a coding tree unit (CTU) in each frame. The saliency map of each frame is divided into CTUs as in HEVC. Experiments are performed under common test conditions with random access (RA) configuration for quantization parameter values QP=22, 27, 32 and 37 [47]. The performance evaluation of the proposed HEVC compliant PVC scheme is performed in terms of bitrate saving, computational complexity, quality assessment using objective and subjective measures.

#### 3.2.1. Bitrate Reduction and Computational Complexity

Bitrate reduction is computed to gauge the compression efficiency. The bitrate reduction ΔBR between the proposed PVC scheme and the HEVC reference model is computed as,

(7) $$\Delta BR=\frac{R_{Pr}-R_{HM}}{R_{HM}}\times 100,$$

where $R_{Pr}$ and $R_{HM}$ represents the bitrate required to encode video using the proposed PVC scheme and HEVC reference software respectively. A negative value of ΔBR indicates percentage bitrate saving achieved by the proposed scheme in comparison with HEVC. Encoding time is used to measure the computational complexity of the proposed PVC scheme in comparison with HEVC. Computational complexity is computed as,

(8) $$\Delta T=\frac{T_{Pr}-T_{HM}}{T_{HM}}\times 100,$$

where $T_{Pr}$ and $T_{HM}$ represents encoding times of video coding using proposed PVC scheme and HEVC reference software respectively. A positive value of ΔT indicates a percentage increase in encoding time by the proposed PVC as compared to HEVC reference software. The encoding time is measured on a computer system with Intel 3.6 GHz quadcore processor, 16 GB RAM.

The proposed PVC scheme is compared with HEVC reference software (HM 16.11) in terms of bitrate saving and encoding time and results are summarized in Table 3. It is evident that the proposed PVC achieves highest bitrate saving at QP=22. An average bitrate saving for sixteen video sequences at QP=22 is 10.37% with maximum 20.08% bitrate saving for video sequence RaceHorses. However, the coding complexity increased by 2.96%. At QP=27, the average bitrate saving for sixteen videos is 6.68%, with a maximum bitrate saving 11.67% achieved by video sequence RaceHorses. However, the coding complexity increased by 2.97%. The average bitrate saving for all video sequences at QP=32 is 5.12%, with a maximum bitrate saving of 9.69% achieved by video sequence Jockey. The coding complexity increased at QP=32 is 3.46%. Whereas the average bitrate saving for sixteen video sequences at QP=37 is 4.10% with a maximum bitrate saving of 7.80% for video sequence Jockey. The coding complexity increase is 3.99% at QP=37. The proposed PVC achieves an average bitrate reduction of 6.57% as compared to the HEVC reference software. This shows a superior performance of the proposed PVC scheme when compared with HEVC reference software.

#### 3.2.2. Objective and Subjective Quality Assessment

An objective evaluation of the proposed scheme was performed by two metrics—multiscale structural similarity index (MS−SSIM) [48] and perceptual peak signal to noise ratio (PPSNR) [49]. MS-SSIM takes into account the mechanism of processing in the early vision system and implements it on multiple scales. The MS−SSIM index between original and distorted videos is computed as,

(9) $$MS-SSIM={\left[l_{M}\left(Orig,Dist\right)\right]}^{\alpha M}\prod_{j=1}^{M}\left[c_{j}{\left(Orig,Dist\right)}^{\beta_{j}}\right]\left[s_{j}{\left(Orig,Dist\right)}^{\gamma_{j}}\right],$$

where $l_{M}\left(Orig,Dist\right)$ denotes luminance comparison, while $c_{j}\left(Orig,Dist\right)$ and $s_{j}\left(Orig,Dist\right)$ represent contrast and structure comparisons at *j*-th scale of original and distorted videos. As mentioned earlier, removing perceptual redundancy while maintaining visual quality is the primary focus of this work. The proposed PVC framework removes perceptually irrelevant information from non-salient regions, while maintaining the visual quality of salient regions. It is worth measuring the PSNR of only salient regions, where perceived visual quality needs to be preserved. Perceptual peak signal to noise ratio has been used as an objective measure to compute the perceived quality [49], which is calculated as,

(10) $$PPSNR=10\log_{10}\times \frac{255\times 255}{\frac{1}{M\times N}\sum_{x=1}^{M}\sum_{y=1}^{N}{\left(V\left(x,y\right)-V^{{}^{\prime }}\left(x,y\right)\right)}^{2}\times \delta_{t}\left(x,y\right)},$$

where $\delta_{t}\left(x,y\right)=1$ for salient region and $\delta_{t}\left(x,y\right)=0$ for non-salient region of original *V* and decoded $V^{\prime }$ frames.

**Figure 5:**
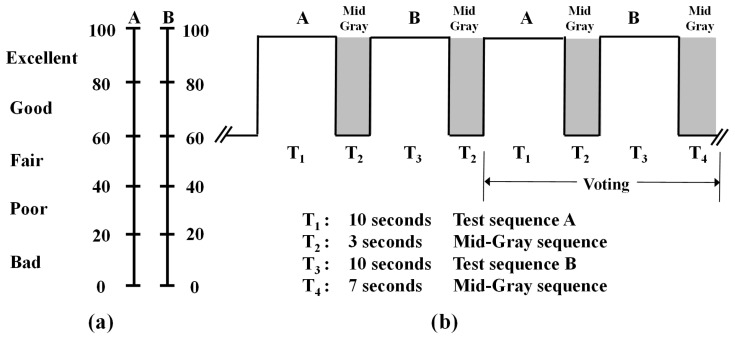
DSCQSMethod. (**a**) Presentation sequence of test and original video sequences. (**b**) Quality-rating form of using continuous scale of DSCQS

Subjective evaluation of the proposed PVC scheme was performed through double stimulus continuous quality scale (DSCQS) [50]. Test and reference videos were shown to the subjects one after the other. The subject compared the visual quality of both the videos and assigned comparative scores to the test and reference videos. Figure 5a shows test and reference video presentation structure in the subjective experiment. Video sequences were randomly ordered with respect to the test and reference for different QP values. To alleviate grading tiredness from session to session, the test sessions were arranged such that the maximum test time taken by each subject was 25 min.

Sixteen subjects (8 males and 8 females) participated in subjective experiments. Display conditions and viewing distance were set according to ITU-R subjective assessment methodology [50]. All subjects were graduate students, aged from 24 to 34 years and were not experts in video coding. For subjective voting, a quality-rating form, as shown in Figure 5b, with continuous scores from 0 to 100 was used. Scores 0 and 100 represent the worst and the best visual qualities, respectively. Subjects observed the overall quality of video sequences and inserted a mark on a grading scale. Mean opinion score (MOS) at each QP for each video sequence was computed by taking an average of the opinion scores of all subjects. For subjective comparison, a difference mean opinion score (DMOS) is computed as,

(11) $$DMOS=MOS_{Pr}-MOS_{HM},$$

where $MOS_{Pr}$ and $MOS_{HM}$ are the mean opinion scores of the video sequences encoded by proposed PVC and HEVC, respectively. A DMOS value close to zero shows that the perceived visual quality of the videos encoded by proposed PVC is as good as that of the HEVC reference software.

**Figure 6:**
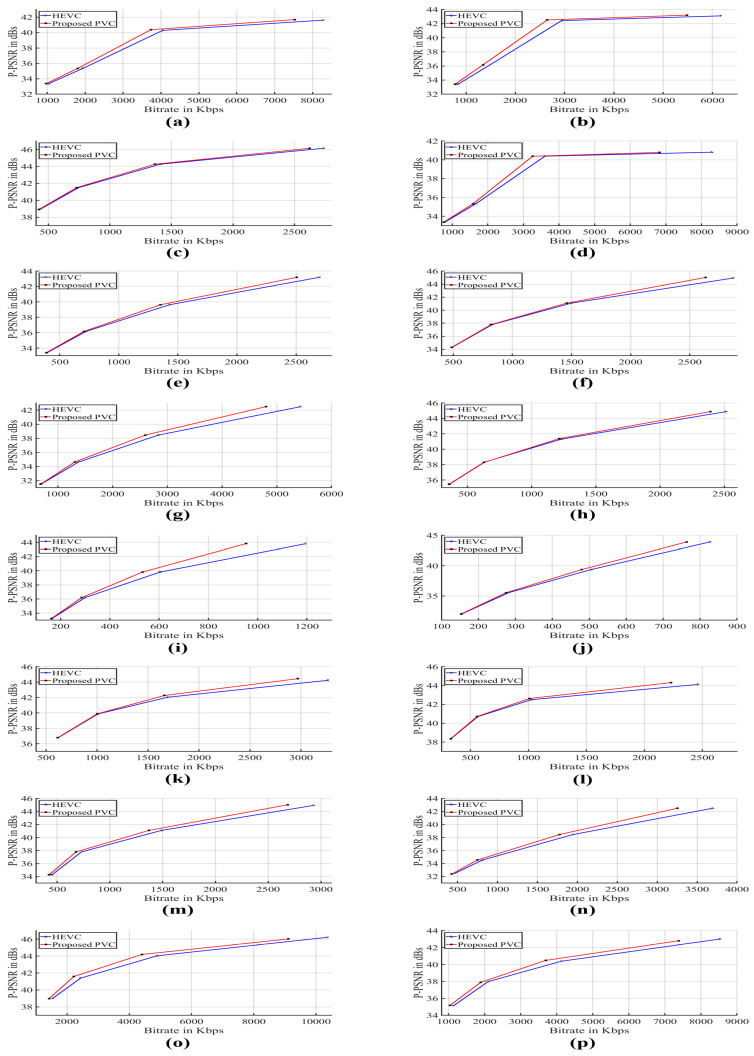
Performance comparison of the proposed PVC scheme and HEVC reference software in terms of bitrate and PPSNR for video sequences (**a**) Nebuta (**b**) SteamLocomotive (**c**) BasketballDrive (**d**) ParkScene (**e**) RaceHorses (**f**) BQMall (**g**) PartyScene (**h**) BasketballDrill (**i**) RaceHorses (**j**) BlowingBubbles (**k**) FourPeople (**l**) Johnny (**m**) BasketballDrillText (**n**) SlideShow (**o**) Bosphorus (**p**) Jockey.

Table 4 summarizes MS−SSIM, PPSNR and DMOS results for ten test video sequences at QP 22, 27, 32 and 37. A negative value of MS−SSIM shows a drop in the values of MS−SSIM. It is evident that the average drop in MS−SSIM for sixteen videos encoded by the proposed PVC scheme is 0.367% in comparison with HEVC. Such a minute difference in MS−SSIM value does not produce a noticeable visible difference. The average PPSNR difference between the proposed PVC and HEVC is 0.019, which signifies that the proposed PVC scheme preserves the visual quality in the salient regions. The average DMOS value of −0.107 is observed for sixteen video sequences, which is not significantly different. This shows that the visual quality of the proposed PVC scheme as perceived by subjects is same as the HEVC reference software but at a lower data rate. A comparison of our proposed HEVC based PVC and HEVC in terms of bitrate and PPSNR is also shown in Figure 6. It is evident that our proposed PVC scheme performs better than the HEVC reference software scheme for all the video sequences used in this work.

**Figure 7:**
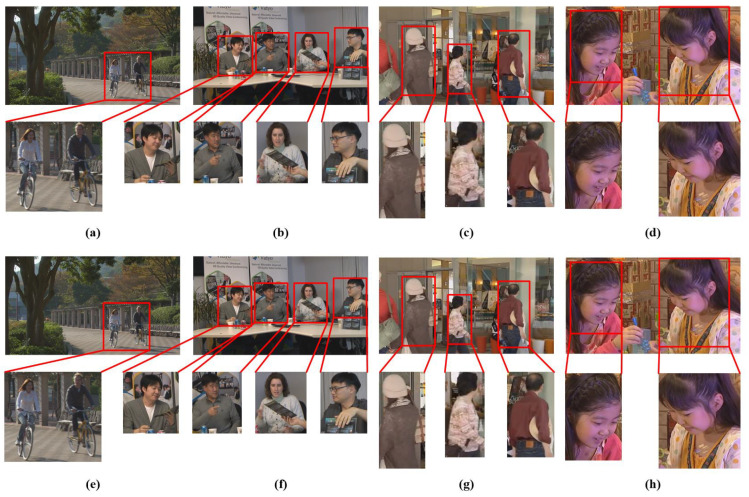
The decoded frames with highlighted salient region of video sequences (**a**) ParkScene, (**b**) FourPeople, (**c**) BQMall and (**d**) BlowingBubbles encoded at QP=22 using HEVC reference software and the decoded frames with highlighted salient region of video sequences (**e**) ParkScene, (**f**) FourPeople, (**g**) BQMall and (**h**) BlowingBubbles encoded video at QP=22 using proposed PVC scheme.

Figure 7 shows the decoded frames of ParkScene, FourPeople, BQMall and BlowingBubbles video sequences at QP=22 using the HEVC reference software and proposed PVC scheme. It is evident that the proposed HEVC compliant entropy based PVC has the same visual quality for visually salient regions in the decoded frame as compared to the reference HEVC encoder but with a reduction in data rate by 17.69% for ParkScene, 8.95% for FourPeople, 8.08% for BQMall and 7.63% for BlowingBubbles.

The comparison of perceptual video coding schemes available in the literature is a challenging task as each scheme utilizes a different set of video sequences and quality evaluation metrics. For example, Sehwan [51] used six video sequences for evaluation and compared results with HEVC HM 16.17. Similarly, Bae [17] used six video sequences and compared results with HEVC HM 11.0. Table 5 presents a comparison in terms of bitrate reduction and DMOS for the video sequences that are common among the proposed, Sehwan [51] and Bae [17] PVC schemes. It is evident that the proposed PVC scheme achieves more bit rate reduction as compared to Bae [17] PVC schemes when encoded at QP=32 and QP=37. This shows that the proposed scheme performs well at low data rates. Similarly, the proposed PVC scheme achieves more bit rate reduction as compared to the Sehwan [51] PVC scheme when encoded at QP=22 and QP=37, which shows better performance of the proposed scheme at low and high data rates. The proposed PVC scheme DMOS values are close to zero for all QP values when compared with both the PVC schemes. This shows that the proposed PVC scheme achieves the same perceived quality with more bit rate saving.

## 4. Conclusions

In this paper, a new HEVC compliant PVC scheme is proposed. An information maximization based visual saliency model was utilized to identify the salient and non-salient regions in each video frame. The perceptual significance of each CTU in a frame was figured out by considering the number of salient and non-salient pixels. A QP value for each CTU was selected in an optimum way based on their perceptual relevance. As a result, fewer bits were assigned to non-salient CTUs in a frame. The proposed PVC scheme was incorporated in HEVC reference implementation HM 16.11. Sixteen test video sequences belonging to Class A, B, C, D, E, F and 4K were encoded using random access configuration. Objective and subjective evaluations were performed to measure the efficacy of the proposed PVC scheme. The proposed HEVC compliant PVC scheme achieves 10.37% of average bitrate reduction at QP=22 for all video sequences, while preserving the perceived visual quality. However, performance improvement costs a nominal increase in computational complexity of the encoder.

## Figures and Tables

**Table 1:** Test Video Sequences.

Class	Video Sequence	Spatial Resolution	Frame Count	Frame Rate
A	Nebuta	2560 × 1600	300	60
A	SteamLocomotive	2560 × 1600	300	60
B	BasketballDrive	1920 × 1080	500	50
B	ParkScene	1920 × 1080	240	24
C	RaceHorses	832 × 480	300	30
C	BQMall	832 × 480	600	60
C	PartyScene	832 × 480	500	50
C	BasketballDrill	832 × 480	500	50
D	RaceHorses	416 × 240	300	30
D	BlowingBubbles	416 × 240	500	50
E	FourPeople	1280 × 720	600	60
E	Johnny	1280 × 720	600	60
F	BasketballDrillText	832 × 480	500	50
F	SlideShow	1280 × 720	500	20
4K	Bosphorus	3840 × 2160	600	120
4K	Jockey	3840 × 2160	600	120

**Table 2:** Performance comparison of entropy based visual saliency algorithms in terms of average precision, recall and F-measure when thresholded at $Th_{o}=9$.

Visual Saliency Model	Precision	Recall	F-Measure
AIM [37]	0.851	0.738	0.790
SSM [41]	0.349	0.828	0.491
EOS [42]	0.357	0.785	0.490
FEMLT [43]	0.326	0.693	0.443

**Table 3:** Performance comparison of the proposed perceptual video coding (PVC) scheme with HEVC in terms of bitrate reduction and encoding time.

Video	QP=22						QP=27					
	Datarate in Kbps			Execution Time in Seconds			Datarate in Kbps			Execution Time in Seconds		
	${\mathrm{HEVC}}_{\mathrm{HM}}$	${\mathrm{PVC}}_{\mathrm{Pr}}$	ΔBR	${\mathrm{HEVC}}_{\mathrm{HM}}$	${\mathrm{PVC}}_{\mathrm{Pr}}$	ΔT	${\mathrm{HEVC}}_{\mathrm{HM}}$	${\mathrm{PVC}}_{\mathrm{Pr}}$	ΔBR	${\mathrm{HEVC}}_{\mathrm{HM}}$	${\mathrm{PVC}}_{\mathrm{Pr}}$	ΔT
**Nebuta** Class A, (2560 × 1600)	8273.54	7530.27	−8.98	615.43	624.36	1.45	4045.32	3735.82	−7.65	579.97	587.88	1.36
**SteamLocomotive** Class A, (2560 × 1600)	6167.96	5483.52	−11.10	604.76	611.89	1.18	2937.66	2648.242	−9.85	584.66	591.23	1.12
**BasketballDrive** Class B, (1920 × 1080)	2735.02	2621.11	−4.16	436.98	442.22	1.20	1411.63	1368.20	−3.08	389.59	401.38	3.03
**ParkScene** Class B, (1920 × 1080)	8284.99	6819.50	−17.69	469.80	481.23	2.43	3606.23	3260.44	−9.59	376.31	391.10	3.93
**RaceHorses** Class C, (832 × 480)	2694.51	2505.65	−7.01	129.27	131.65	1.84	1429.59	1352.99	−5.36	105.54	108.04	2.37
**BQMall** Class C, (832 × 480)	2866.08	2634.54	−8.08	83.48	91.01	9.03	1499.76	1446.73	−3.54	71.22	74.25	4.25
**PartyScene** Class C, (832 × 480)	5429.73	4804.70	−11.51	114.32	115.99	1.46	2833.48	2595.78	−8.39	95.90	97.09	1.24
**BasketballDrill** Class C, (832 × 480)	2511.80	2371.85	−5.57	95.85	101.12	5.50	1235.53	1205.83	−2.40	78.98	81.75	3.51
**RaceHorses** Class D, (416 × 240)	1193.70	954.00	−20.08	33.61	34.36	2.24	603.53	533.07	−11.67	27.09	29.01	7.09
**BlowingBubbles** Class D, (416 × 240)	826.60	763.50	−7.63	16.25	16.99	4.53	504.90	479.40	−5.05	14.63	15.13	3.39
**FourPeople** Class E, (1280 × 720)	3262.20	2970.21	−8.95	144.35	149.52	3.58	1692.81	1660.50	−1.91	129.69	132.87	2.45
**Johnny** Class E, (1280 × 720)	2461.98	2228.79	−9.47	149.62	151.18	1.04	1024.89	1009.11	−1.54	127.18	129.60	1.90
**BasketballDrillText** Class F, (832 × 480)	2929.41	2685.74	−8.32	114.52	118.39	3.38	1488.25	1369.92	−7.95	98.34	101.46	3.17
**SlideShow** Class F, (1280 × 720)	3688.73	3251.62	−11.85	152.73	156.75	2.63	1942.56	1778.11	−8.47	121.89	125.78	3.19
**Bosphorus** 4K, (3840 × 2160)	10,367.34	9108.77	−12.14	986.33	998.54	1.24	4898.65	4406.66	−10.04	902.42	912.70	1.14
**Jockey** 4K, (3840 × 2160)	8522.09	7382.45	−13.37	979.25	992.38	1.34	4122.54	3691.52	−10.46	899.77	909.61	1.09
**Average**			**−10.37**			**2.96**			**−6.68**			**2.97**
**Video**	QP=32						QP=37					
	**Datarate in Kbps**			**Execution Time in Seconds**			**Datarate in Kbps**			**Execution Time in Seconds**		
	${\mathrm{HEVC}}_{\mathrm{HM}}$	${\mathrm{PVC}}_{\mathrm{Pr}}$	ΔBR	${\mathrm{HEVC}}_{\mathrm{HM}}$	${\mathrm{PVC}}_{\mathrm{Pr}}$	ΔT	${\mathrm{HEVC}}_{\mathrm{HM}}$	${\mathrm{PVC}}_{\mathrm{Pr}}$	ΔBR	${\mathrm{HEVC}}_{\mathrm{HM}}$	${\mathrm{PVC}}_{\mathrm{Pr}}$	ΔT
**Nebuta** Class A, (2560 × 1600)	1936.38	1798.56	−7.12	556.82	563.96	1.28	1038.88	972.49	−6.39	529.44	535.84	1.21
**SteamLocomotive** Class A, (2560 × 1600)	1488.45	1345.26	−9.62	541.75	547.56	1.07	834.64	773.84	−7.28	519.63	524.62	0.96
**BasketballDrive** Class B, (1920 × 1080)	746.62	729.32	−2.32	348.82	356.21	2.12	432.17	421.54	−2.46	319.04	337.32	5.73
**ParkScene** Class B, (1920 × 1080)	1669.40	1587.44	−4.91	326.31	341.22	4.57	792.64	768.59	−3.03	296.06	306.06	3.38
**RaceHorses** Class C, (832 × 480)	723.77	707.04	−2.31	87.60	89.33	1.97	395.37	388.12	−1.83	75.24	79.12	5.15
**BQMall** Class C, (832 × 480)	830.85	812.59	−2.20	65.15	70.91	8.84	488.82	475.40	−2.75	58.91	61.24	3.96
**PartyScene** Class C, (832 × 480)	1379.75	1310.73	−5.00	76.29	78.82	3.32	703.90	684.93	−2.70	62.64	64.71	3.31
**BasketballDrill** Class C, (832 × 480)	630.00	614.10	−2.52	67.80	70.34	3.74	360.58	352.75	−2.17	62.21	66.44	6.79
**RaceHorses** Class D, (416 × 240)	299.90	285.74	−4.72	22.01	23.68	7.59	165.11	161.60	−2.12	18.65	19.78	6.09
**BlowingBubbles** Class D, (416 × 240)	282.38	271.83	−3.74	13.78	14.09	2.24	154.78	151.01	−2.43	12.92	13.64	5.53
**FourPeople** Class E, (1280 × 720)	1010.52	989.04	−2.13	123.37	127.43	3.29	619.92	607.62	−1.98	120.30	124.96	3.87
**Johnny** Class E, (1280 × 720)	563.64	551.97	−2.07	118.48	122.58	3.46	332.31	325.61	−2.02	115.17	120.55	4.67
**BasketballDrillText** Class F, (832 × 480)	732.76	680.68	−7.11	86.12	88.46	2.72	451.89	424.03	−6.17	72.78	74.56	2.45
**SlideShow** Class F, (1280 × 720)	811.33	750.45	−7.50	110.34	112.85	2.27	460.81	429.31	−6.84	97.54	100.28	2.81
**Bosphorus** 4K, (3840 × 2160)	2438.52	2217.94	−9.05	881.15	890.45	1.06	1542.44	1424.17	−7.67	854.34	862.65	0.97
**Jockey** 4K, (3840 × 2160)	2093.55	1890.78	−9.69	861.02	869.93	1.03	1127.29	1039.41	−7.80	837.88	846.12	0.98
**Average**			**−5.12**			**3.46**			**−4.10**			**3.99**

**Table 4:** Performance comparison of the proposed PVC scheme with HEVC in terms of objective (MS-SSIM and PPSNR)and subjective (DMOS) measures.

Video	QP	MS−SSIM			PPSNR			DMOS
		${\mathrm{HEVC}}_{\mathrm{HM}}$	${\mathrm{PVC}}_{\mathrm{Pr}}$	$\Delta_{\mathrm{MS}-\mathrm{SSIM}}$	${\mathrm{HEVC}}_{\mathrm{Pr}}$	${\mathrm{PVC}}_{\mathrm{Pr}}$	$\Delta_{\mathrm{PPSNR}}$	
**Nebuta** Class A (2560 × 1600)	22	0.992	0.994	0.192	41.614	41.691	0.077	0.07
	27	0.992	0.991	−0.040	40.289	40.384	0.095	0.20
	32	0.987	0.986	−0.091	36.472	36.384	−0.087	0.13
	37	0.981	0.979	−0.133	33.371	33.380	0.009	−0.13
**SteamLocomotive** Class A (2560 × 1600)	22	0.997	0.994	−0.248	43.102	43.203	0.101	0.20
	27	0.993	0.989	−0.357	42.430	42.529	0.099	−0.13
	32	0.989	0.996	0.749	36.171	36.170	−0.001	−0.53
	37	0.983	0.974	−0.851	33.405	33.403	−0.002	−0.60
**BasketballDrive** Class B (1920 × 1080)	22	0.996	0.994	−0.171	46.145	46.143	−0.002	0.13
	27	0.992	0.990	−0.232	44.267	44.266	−0.001	−0.13
	32	0.985	0.982	−0.304	41.496	41.494	−0.002	−0.20
	37	0.974	0.969	−0.472	38.918	38.917	−0.001	−0.07
**ParkScene** Class B (1920 × 1080)	22	0.990	0.990	−0.077	40.814	40.791	−0.023	0.20
	27	0.983	0.982	−0.065	40.391	40.384	−0.007	0.07
	32	0.969	0.968	−0.047	35.339	35.337	−0.002	0.07
	37	0.944	0.944	−0.034	33.371	33.380	0.009	−0.13
**RaceHorses** Class C (832 × 480)	22	0.995	0.989	−0.583	43.192	43.192	0.000	0.27
	27	0.989	0.982	−0.728	39.630	39.629	−0.001	−0.20
	32	0.976	0.947	−3.022	36.171	36.170	−0.001	0.07
	37	0.954	0.947	−0.786	33.405	33.403	−0.002	−0.33
**BQMall** Class C (832 × 480)	22	0.997	0.996	−0.167	44.933	45.034	0.101	0.13
	27	0.994	0.992	−0.182	41.087	41.092	0.005	−0.07
	32	0.988	0.986	−0.223	37.799	37.791	−0.008	−0.33
	37	0.976	0.974	−0.258	34.281	34.278	−0.003	−0.47
**PartyScene** Class C (832 × 480)	22	0.996	0.986	−1.074	42.480	42.492	0.012	0.33
	27	0.991	0.978	−1.241	38.452	38.458	0.006	0.13
	32	0.976	0.963	−1.375	34.644	34.653	0.009	0.07
	37	0.950	0.938	−1.302	31.552	31.493	−0.059	−0.07
**BasketballDrill** Class C (832 × 480)	22	0.995	0.994	−0.097	44.879	44.884	0.005	0.13
	27	0.990	0.989	−0.105	41.321	41.379	0.058	0.00
	32	0.981	0.979	−0.158	38.299	38.290	−0.009	−0.60
	37	0.964	0.962	−0.255	35.459	35.444	−0.015	−0.53
**RaceHorses** Class D (416 × 240)	22	0.995	0.989	−0.614	43.809	43.844	0.035	0.07
	27	0.988	0.980	−0.863	39.811	39.822	0.011	0.20
	32	0.974	0.965	−0.921	36.209	36.201	−0.008	0.13
	37	0.948	0.938	−1.130	33.227	33.224	−0.003	−0.13
**BlowingBubbles** Class D (416 × 240)	22	0.998	0.995	−0.255	43.908	43.911	0.003	0.13
	27	0.994	0.991	−0.225	39.366	39.364	−0.002	−0.40
	32	0.985	0.983	−0.148	35.562	35.540	−0.022	−0.53
	37	0.967	0.965	−0.207	32.022	32.021	−0.001	−0.60
**FourPeople** Class E (1280 × 720)	22	0.995	0.995	−0.026	44.239	44.455	0.216	0.13
	27	0.993	0.993	−0.022	42.042	42.270	0.229	−0.27
	32	0.989	0.989	0.000	39.883	39.884	0.001	−0.13
	37	0.982	0.982	0.013	36.802	36.781	−0.021	−0.40
**Johnny** Class E (1280 × 720)	22	0.993	0.993	−0.028	44.122	44.318	0.196	0.07
	27	0.991	0.991	−0.009	42.502	42.622	0.120	−0.27
	32	0.987	0.987	0.003	40.700	40.715	0.016	−0.13
	37	0.981	0.981	0.012	38.351	38.347	−0.003	−0.60
**BasketballDrillText** Class F (832 × 480)	22	0.992	0.991	−0.082	44.933	45.034	0.101	0.13
	27	0.990	0.989	−0.094	41.087	41.092	0.005	0.07
	32	0.986	0.985	−0.139	37.799	37.791	−0.008	−0.07
	37	0.972	0.970	−0.187	34.281	34.278	−0.003	−0.53
**SlideShow** Class F (1280 × 720)	22	0.996	0.994	−0.203	42.533	42.572	0.039	0.20
	27	0.994	0.992	−0.231	39.165	39.169	0.004	−0.07
	32	0.986	0.982	−0.411	35.626	35.644	0.018	−0.33
	37	0.982	0.977	−0.442	32.452	32.393	−0.059	−0.47
**Bosphorus** 4K (3840 × 2160)	22	0.993	0.992	−0.058	46.214	46.021	−0.193	0.20
	27	0.991	0.990	−0.120	44.035	44.191	0.156	0.13
	32	0.987	0.985	−0.220	41.398	41.586	0.188	-0.07
	37	0.978	0.976	−0.274	39.017	38.981	−0.036	−0.53
**Jockey** 4K (3840 × 2160)	22	0.995	0.991	−0.354	43.011	42.813	−0.198	0.13
	27	0.991	0.985	−0.687	40.389	40.488	0.099	0.00
	32	0.988	0.979	−0.869	37.982	37.901	−0.081	−0.13
	37	0.979	0.969	−0.971	35.110	35.183	0.073	−0.53
**Average**		**0.985**	**0.981**	**−0.367**	**39.262**	**39.281**	**0.019**	**−0.107**

**Table 5:** Performance comparison of the proposed PVC with Sehwan [51] and Bae [17] in terms of bitrate reduction and DMOS values for common videos.

Video	QP	Sehwan [51]		Bae [17]		${\mathrm{PVC}}_{\mathrm{Pr}}$	
		ΔBR	DMOS	ΔBR	DMOS	ΔBR	DMOS
**ParkScene** Class B (1920 × 1080)	22	−12.39	−1.00	−21.10	2.00	−17.69	0.2
	27	−13.52	−0.90	−6.00	−1.20	−9.59	0.07
	32	−6.23	−0.10	−0.80	0.00	−4.91	0.07
	37	−0.43	0.40	0.00	−0.10	−3.03	−0.13
**BQMall** Class C (832 × 480)	22	−2.75	−0.60	−17.5	1.70	−8.08	0.13
	27	−10.43	−0.20	−5.60	−1.10	−3.54	−0.07
	32	−8.43	−1.00	−0.30	−0.20	−2.2	−0.33
	37	−1.78	0.10	−0.30	0.10	−2.75	−0.47
**RaceHorses** Class C (832 × 480)	22	−15.53	−0.20	−27.40	1.20	−7.01	0.27
	27	−14.78	−0.80	−10.40	−0.80	−5.36	−0.2
	32	−9.42	0.40	−1.10	0.50	−2.31	0.07
	37	−1.86	0.00	−0.10	1.10	−1.83	−0.33
**PartyScene** Class C (832 × 480)	22	−6.23	−0.20	−26.70	0.30	−11.51	0.33
	27	−14.93	−0.60	−9.70	1.10	−8.39	0.13
	32	−13.69	−0.10	−1.50	0.10	−4.91	0.07
	37	−2.95	−0.60	−0.40	−0.10	−3.03	−0.07
